# Hemodialysis vascular sound index as a diagnostic tool for vascular access stenosis: a prospective matched observational study

**DOI:** 10.1186/s12882-026-04815-2

**Published:** 2026-02-21

**Authors:** Yasumasa Hitomi, Naoki Suzuki, Toshiya Enmeiji, Hiroya Takata, Takahiro Konishi, Yuki Takeda, Yuta Masugi, Masato Nishimura, Nodoka Sato

**Affiliations:** 1 Division of Clinical Engineering, Tojinkai Hospital, Kyoto, Japan; 2 Division of Data Science, Tojinkai Hospital, Kyoto, Japan; 3 Cardiovascular Division, Tojinkai Satellite Clinic, Kyoto, Japan; 4 Urology Division, Tojinkai Hospital, Kyoto, Japan

**Keywords:** Hemodialysis, Vascular access, Arteriovenous fistula, Vascular access stenosis, Ultrasound surveillance, Vascular sound analysis

## Abstract

**Background:**

Vascular access (VA) monitoring is critical for hemodialysis patients. While vascular ultrasound (US) provides high diagnostic accuracy for VA stenosis, its reliance on equipment and trained operators can limit routine implementation. The Hemodialysis Vascular Sound Index (HVSI), derived from vascular murmur analysis, may provide a simple, objective adjunct for triaging patients who require confirmatory US and/or vascular access interventional treatment (VAIVT).

**Objectives:**

This study evaluated the diagnostic performance of HVSI for detecting VA stenosis and reduced brachial artery flow volume (FV), and examined its association with US-based parameters including resistance index (RI).

**Methods:**

This prospective matched observational study included 202 hemodialysis patients: 101 with clinically significant stenosis requiring VAIVT (study group) and 101 ultrasound-confirmed stable controls (control group). Participants were matched by age, sex, dialysis duration, diabetes status, Kt/V, and blood data using propensity score matching. HVSI was measured using an electronic stethoscope placed over the anastomosis before dialysis. FV and RI were assessed using Doppler US. Diagnostic performance was evaluated using receiver operating characteristic (ROC) analyses, including sensitivity, specificity, and area under the curve (AUC). A small verification cohort (*n* = 20) was also analyzed to explore the reproducibility of predefined HVSI cutoffs.

**Results:**

HVSI showed significant correlation with FV (R^2^ = 0.58, *p* < 0.001) and inverse correlation with RI (R^2^ = 0.32, *p* < 0.001). For FV thresholds ≤500, ≤400, and ≤350 mL/min, HVSI showed sensitivities of 86.3–94.4%, specificities of 78.7–82.9%, and AUCs of 0.90–0.94. Diagnostic accuracy tended to be higher in non-bifurcated vessels. In the verification cohort, predefined HVSI cutoffs showed high specificity for FV < 400 mL/min and strong concordance for identifying VAIVT necessity.

**Conclusion:**

HVSI demonstrated clinically meaningful diagnostic accuracy for reduced FV and VA stenosis in this matched observational cohort. However, because this design compared clinically evident stenosis cases with ultrasound-confirmed stable controls, diagnostic performance may be overestimated relative to consecutive real-world screening populations. Therefore, HVSI should be interpreted not as a replacement for vascular ultrasound, but as a simple screening adjunct to triage patients who require confirmatory ultrasound and/or VAIVT. Further studies in consecutively enrolled cohorts are warranted to validate generalizability and optimize implementation.

**Supplementary information:**

The online version contains supplementary material available at 10.1186/s12882-026-04815-2.

## Introduction

Vascular access (VA) serves as a critical lifeline for hemodialysis patients, and effective monitoring is essential for its long-term maintenance. Routine monitoring commonly includes inspection, palpation, and auscultation, whereas surveillance typically relies on imaging such as vascular ultrasound (US). US is widely recognized as a reliable tool for diagnosing VA stenosis, with reported sensitivity and specificity of 82–96% and 76–97%, respectively [1, 2]. Despite its diagnostic performance, routine US surveillance may be challenging in some clinical settings due to limitations in equipment availability, staffing, and the need for trained operators.

In addition to physical examination, several monitoring and surveillance approaches have been proposed to detect access dysfunction earlier, including hemodialysis-based access blood flow monitoring (e.g., Delta-H method) and guideline-based surveillance strategies. These approaches can help identify patients who may benefit from confirmatory imaging and timely intervention, although implementation and resource requirements vary across settings [3, 4].

Auscultation offers a simple and non-invasive alternative. However, conventional auscultation using analog stethoscopes relies on subjective perception and experience, resulting in variability across individuals and time points. This subjectivity limits the utility of auscultation for standardized monitoring or longitudinal comparisons. To address these limitations, electronic stethoscopes and signal processing methods have been explored to quantify vascular sounds [5–8]. A recent pilot study using a new-generation digital stethoscope also supported the feasibility of objective sound-based assessment in hemodialysis vascular access management [9]. Furthermore, machine-learning approaches, including deep learning–based analysis of blood flow sounds, have been reported as promising tools for detecting AVF stenosis [10]. Most prior studies, however, have been exploratory and relatively small.

**Fig. 1:**
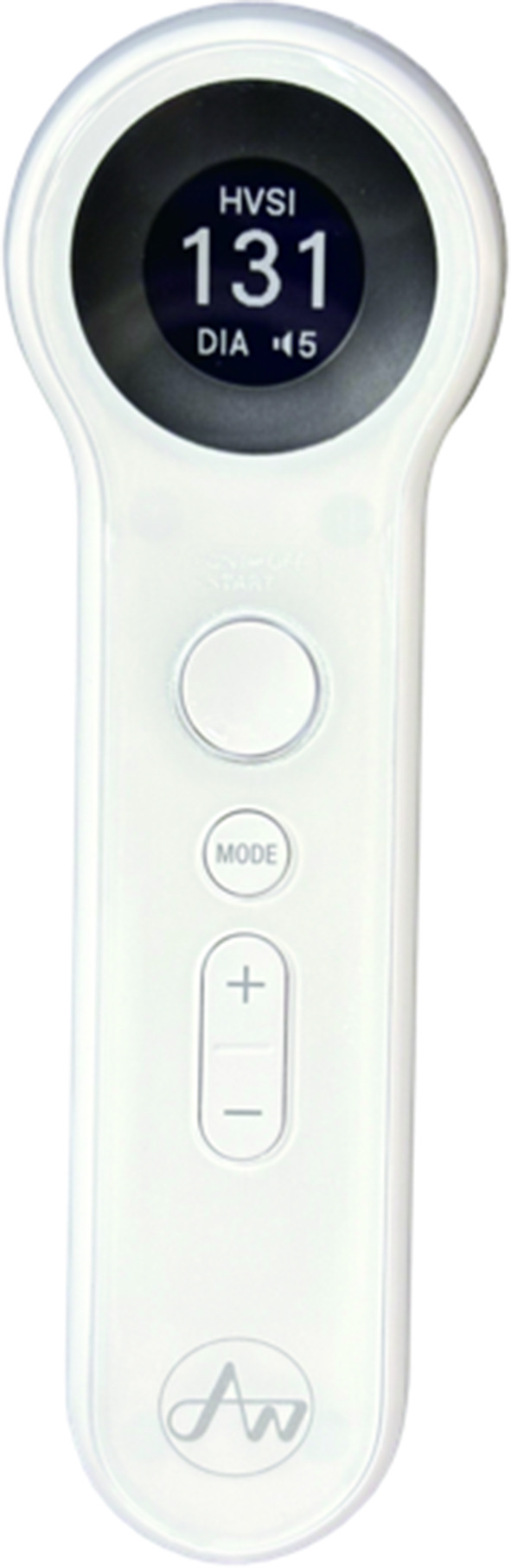
Conceptual schematic of the hemodialysis vascular sound index (HVSI). HVSI is calculated from vascular sound signals recorded at the arteriovenous fistula anastomosis using an electronic stethoscope and converted into a 0–999 score

Recently, the Hemodialysis Vascular Sound Index (HVSI), an algorithm-based index derived from vascular sound analysis, has been proposed as an objective indicator for functional VA assessment [11]. The HVSI used in this study is an enhanced version of previously developed vascular sound systems [11], incorporating improved analytical parameters to strengthen correlation with hemodynamic indices (Fig. 1). Preliminary studies reported an association between HVSI and brachial artery flow volume (FV) [11, 12], but its diagnostic accuracy—particularly in the presence of vessel bifurcation—remains insufficiently characterized.

The aim of this prospective matched observational study was to evaluate the diagnostic performance of HVSI for VA stenosis and reduced FV in hemodialysis patients. We examined correlations between HVSI and ultrasound parameters including FV and resistance index (RI), determined HVSI cutoffs for detecting clinically relevant reduced FV, and assessed HVSI performance in cases with and without vessel bifurcation. Because HVSI is intended to support routine screening, we also explored reproducibility of predefined cutoffs in a small verification cohort.

## Materials and methods

### Study design and setting

This was a single-center prospective matched observational study conducted at Tojinkai Hospital (Kyoto, Japan). The study compared patients requiring vascular access interventional treatment (VAIVT) for clinically significant stenosis with ultrasound-confirmed stable controls. Because case identification and control selection were based on clinically evident stenosis and stable VA controls, this design may overestimate diagnostic performance compared with consecutively enrolled screening populations; this limitation is acknowledged in the Abstract and Discussion.

### Study patients

Adult patients who underwent maintenance hemodialysis and VA evaluation between February 1, 2023 and March 31, 2024 were eligible. The study group included patients diagnosed with VA stenosis requiring VAIVT during the observation period. Stenosis requiring VAIVT was defined as ≥50% diameter reduction by ultrasound plus at least one clinical abnormality (e.g., decreased blood flow, increased venous pressure, prolonged hemostasis) judged by a specialist to require intervention [13]. Patients with arteriovenous grafts (AVGs) were excluded because hemodynamics and acoustic characteristics may differ from native fistulas, and indices such as RI may be less reliable in AVG settings. Patients with complete access occlusion were also excluded because HVSI would be expected to be uniformly low and would not reflect screening performance.

For each stenosis case, one matched control was selected from a pool of ultrasound-confirmed stable VA cases (1:1 matching). “Stable VA” controls were defined as those who met all of the following: (1) ultrasound assessment within the preceding 6 months; (2) FV ≥ 500 mL/min; (3) RI ≤0.60; (4) no diameter reduction ≥ 2.5 mm on ultrasound; and (5) no PTA/VAIVT within the preceding 12 months. Controls were randomly sampled from the eligible stable VA pool after matching.

### Matching procedure

Matching was performed using propensity score matching from an initial cohort of 319 screened patients. Matching variables included age, sex, dialysis duration, diabetes status, Kt/V, and baseline blood data. A 1:1 nearest-neighbor matching approach without replacement was applied to derive the final matched cohort (101 cases and 101 controls).

### Ethics

This study was conducted in accordance with the Declaration of Helsinki. Ethical approval was granted by the Ethics Committee for Human Research at Tojinkai Hospital (approval number: 2022–02). Written informed consent was obtained from all participants prior to enrollment.

This study was retrospectively registered with the University Hospital Medical Information Network Clinical Trials Registry (UMIN-CTR) under trial number UMIN000056433 on December 12, 2024 due to an administrative oversight.

### Hemodialysis Vascular Sound Index (HVSI) measurement

**Fig. 2:**
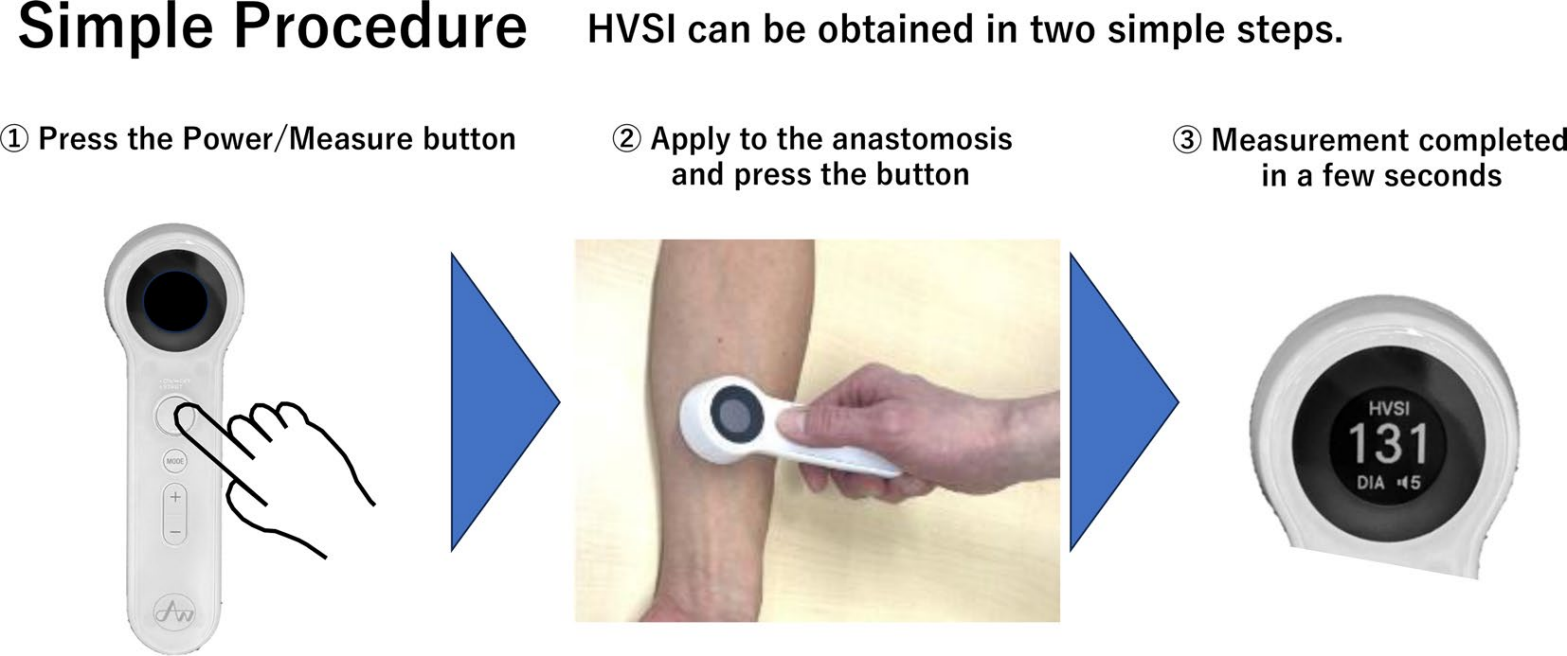
HVSI measurement procedure. The HVSI monitor diaphragm is placed directly over the anastomosis site of the arteriovenous fistula, and recordings are obtained for approximately 5–10 seconds in the resting supine position before dialysis

Vascular sounds were recorded at the arteriovenous anastomosis using an electronic stethoscope incorporated into the HVSI monitor (Air Water Medical Inc., Saitama, Japan). The monitor diaphragm was placed directly over the anastomosis site, and recordings were obtained for approximately 5–10 seconds in a resting supine position before dialysis (Fig. 2). To minimize measurement variability, the anastomosis location was confirmed by ultrasound prior to HVSI recording to ensure accurate placement. Each measurement was repeated three times, and the average value was used for analysis.

Recorded signals were converted into digital data and processed using a short-time Fourier transform to extract frequency-domain characteristics and generate a time-based power spectrum. A band-pass filter (50–1500 Hz) was applied initially, followed by a low-pass filter (cutoff: 300 Hz). Filtered frequency components were converted into absolute values, time-averaged, and normalized to produce an HVSI score ranging from 0 to 999.

### Ultrasonography for vascular access

Ultrasonography was performed using an ultrasound device (Sonimage HS2, Konica Minolta, Inc., Tokyo, Japan). Mean brachial artery FV and RI were measured on the same day before dialysis. FV was calculated as follows:

1 $$\begin{aligned}FV \left( {mL/\min } \right) &= Vessel Area \left( {cm^{2}} \right) \times Time\cr&\quad- Averaged Velocity \left( {{\rm{m}}/{\rm{s}}} \right) \times 60 \left( {\rm{s}} \right)\end{aligned}$$

where

2 $$\begin{aligned}Time - Averaged\,Velocity =& Mean\,Time\,Integral\\&/Heart\,Rate\end{aligned}$$

RI was calculated as:

3 $$\begin{aligned}RI =& \left( {Peak\,Systolic\,Velocity - Lowest\,Diastolic\,Velocity} \right) \\&/Peak\,Systolic\,Velocity\end{aligned}$$

Stenosis severity was assessed morphologically using vessel diameter reduction on ultrasound (diameter-based assessment). PSV-based criteria were not used for stenosis definition in this study.

### Definition of vessel bifurcation

Vessel bifurcation was defined as the presence of a branch with a diameter ≥2 mm within the vascular segment extending from the anastomosis to the stenotic lesion. Presence or absence of vessel bifurcation was determined by ultrasound.

### Laboratory measures

Blood samples (10 mL) were collected before hemodialysis to assess biochemical and hematological variables including hemoglobin, urea nitrogen, creatinine, calcium, inorganic phosphorus, sodium, albumin, β2-microglobulin, and C-reactive protein (CRP). Single-pool Kt/V was calculated using the Shinzato method [14].

### Bias minimization and blinding

Evaluators were not blinded to group allocation; however, HVSI recordings were obtained prior to review of ultrasound findings for each case. Furthermore, standardized procedures were used across trained technicians, including ultrasound confirmation of anastomosis location and triplicate HVSI recording with averaging to minimize operator-dependent variability. Remaining risk of measurement bias is discussed as a limitation.

### Statistical analysis

Baseline characteristics were compared between groups using Welch’s t-test or Mann–Whitney U test for continuous variables and χ^2^ test for categorical variables. Associations between HVSI and FV or RI were assessed using simple linear regression and Pearson correlation, respectively.

Diagnostic performance of HVSI for reduced FV was evaluated using ROC analysis. Cutoff values based on FV criteria (≤500, ≤400, and ≤350 mL/min) were derived using the Youden index. ROC analyses were also performed for identifying stenosis requiring VAIVT, including subgroup analyses according to presence or absence of vessel bifurcation.

To explore reproducibility, an additional verification cohort (*n* = 20), randomly selected from ultrasound-confirmed normal VA cases meeting the control criteria, was analyzed to assess concordance between predefined HVSI cutoffs and ultrasound-based FV < 400 mL/min and VAIVT necessity. This exploratory analysis was considered hypothesis-generating.

Normally distributed data are presented as mean ± standard deviation, and non-normally distributed data as median (interquartile range). Statistical significance was defined as *p* < 0.05. Analyses were performed using EZR (Saitama Medical Center, Jichi Medical University, Saitama, Japan; version 1.68), a graphical user interface for R (R Foundation for Statistical Computing, Vienna, Austria; version 4.3.1) [15].

## Results

A total of 202 adult patients were enrolled: 101 in the stenosis/VAIVT group and 101 matched controls with ultrasound-confirmed stable vascular access. All participants completed the study, and no data were excluded from analysis. No adverse events or unintended effects were reported in either group. In the stenosis/VAIVT group, 18 patients had vessel bifurcation between the anastomosis and the stenotic lesion, while 83 had no bifurcation. Additionally, 7 of 101 patients (6.9%) exhibited peri-anastomotic stenosis.

An additional verification cohort (*n* = 20), randomly selected from ultrasound-confirmed normal VA cases, was analyzed to assess the reproducibility of the predefined HVSI cutoffs.

Baseline characteristics are shown in Table 1. In the control and stenosis/VAIVT groups, the mean age was 69.8 and 70.9 years; 64.4% and 60.4% were male; 45.5% and 46.5% had diabetes mellitus; median CRP was 0.10 and 0.30 mg/dL; and 57.4% and 85.1% had a history of VAIVT, respectively. CRP and past VAIVT were significantly higher in the stenosis/VAIVT group than in the control group. The distribution of AVF types differed between groups (*p* = 0.001). Patients were matched on prespecified covariates, but residual imbalance in AVF location remained and was therefore addressed in multivariable analyses.

**Table 1:** Characteristics of the study participants

Variables	Control (*n* = 101)	Study (*n* = 101)	P value
Age, y	69.8 ± 11.9	70.9 ± 12.6	0.511
Male, n (%)	65 (64.4)	61 (60.4)	0.663
Dialysis duration, y	6.0 (3.0–12.0)	7.00 (3.0–12.0)	0.752
Diabetes mellitus, n (%)	46 (45.5)	47 (46.5)	1.000
Dry weight, kg	58.1 ± 14.7	58.0 ± 11.6	0.970
Albumin, g/dL	3.57 ± 0.33	3.48 ± 0.42	0.069
Blood urea nitrogen, mg/dL	63.7 ± 16.3	62.5 ± 15.6	0.607
Calcium, mg/dL	8.7 ± 0.6	8.8 ± 0.7	0.074
Creatinine, mg/dL	10.7 ± 3.0	10.8 ± 2.7	0.832
Hemoglobin, g/dL	10.9 ± 1.3	11.1 ± 1.3	0.551
Inorganic phosphorus, mg/dL	5.1 ± 1.2	5.4 ± 1.4	0.094
C-reactive protein, mg/dL	0.10 (0.00–0.20)	0.30 (0.10–0.70)	<0.001
Past cardiovascular events, n (%)	35 (34.7)	31 (30.7)	0.653
Past endovascular therapy for lower extremity, n (%)	5 (5.0)	5 (5.0)	1.000
Past vascular access interventional therapy, n (%)	58 (57.4)	86 (85.1)	<0.001
Vascular access calcification, n (%)	0 (0)	0 (0)	–
Arteriovenous fistula types			
-Brachial artery, n (%)	11 (10.9)	18 (17.8)	0.001
-Radial artery at the wrist, n (%)	78 (77.2)	54 (53.5)	
-Radial artery at anatomical snuffbox, n (%)	12 (11.9)	29 (28.7)	
Stenosis Location			
-Venous needling site, n (%)	–	13 (12.9)	–
-Peri-anastomotic region, n (%)	–	7 (6.9)	–
-Swing site, n (%)	–	62 (61.4)	–
-Arterial needling site, n (%)	–	13 (12.9)	–
-Outflow veins, n (%)	–	6 (5.9)	–

**Fig. 3:**
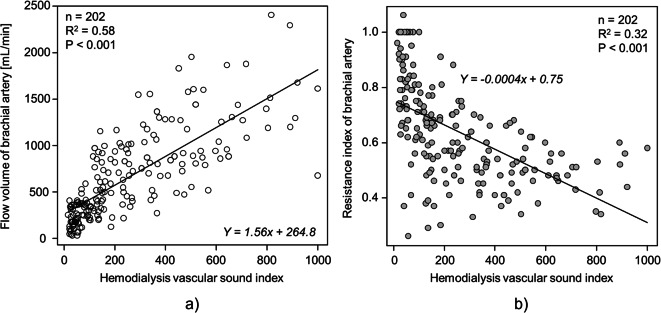
Association between HVSI and ultrasound-derived parameters. Scatterplots showing the relationship of HVSI with brachial artery flow volume (FV) and resistance index (RI)

The relationship between brachial artery flow volume (FV) or resistance index (RI) and the HVSI at the anastomosis is shown in Fig. 3. FV demonstrated a significant positive correlation with HVSI (coefficient of determination R^2^ = 0.58, *p* < 0.001), whereas RI showed a significant negative correlation with HVSI (R^2^ = 0.32, *p* < 0.001).

**Fig. 4:**
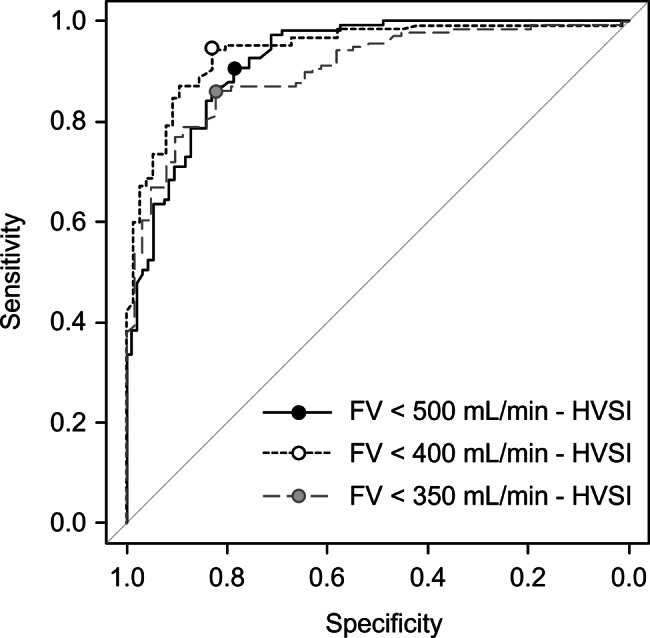
Receiver operating characteristic (ROC) curves for HVSI predicting reduced flow volume. ROC curves are shown for FV thresholds of <500, <400, and <350 mL/min

Receiver operating characteristic (ROC) curves for the HVSI at the anastomosis predicting decreased FV are shown in Fig. 4. When FV < 500 mL/min was considered positive, the HVSI cutoff was 142, with a sensitivity of 90.7%, specificity of 78.7%, and AUC of 0.92 (95% CI: 0.86–0.96). When FV < 400 mL/min was considered positive, the HVSI cutoff was 121, with a sensitivity of 94.4%, specificity of 82.9%, and AUC of 0.94 (95% CI: 0.91–0.98). When FV < 350 mL/min was considered positive, the HVSI cutoff was 121 (same cutoff as FV < 400 mL/min by the Youden index), with a sensitivity of 86.3%, specificity of 82.3%, and AUC of 0.90 (95% CI: 0.86–0.95).

**Fig. 5:**
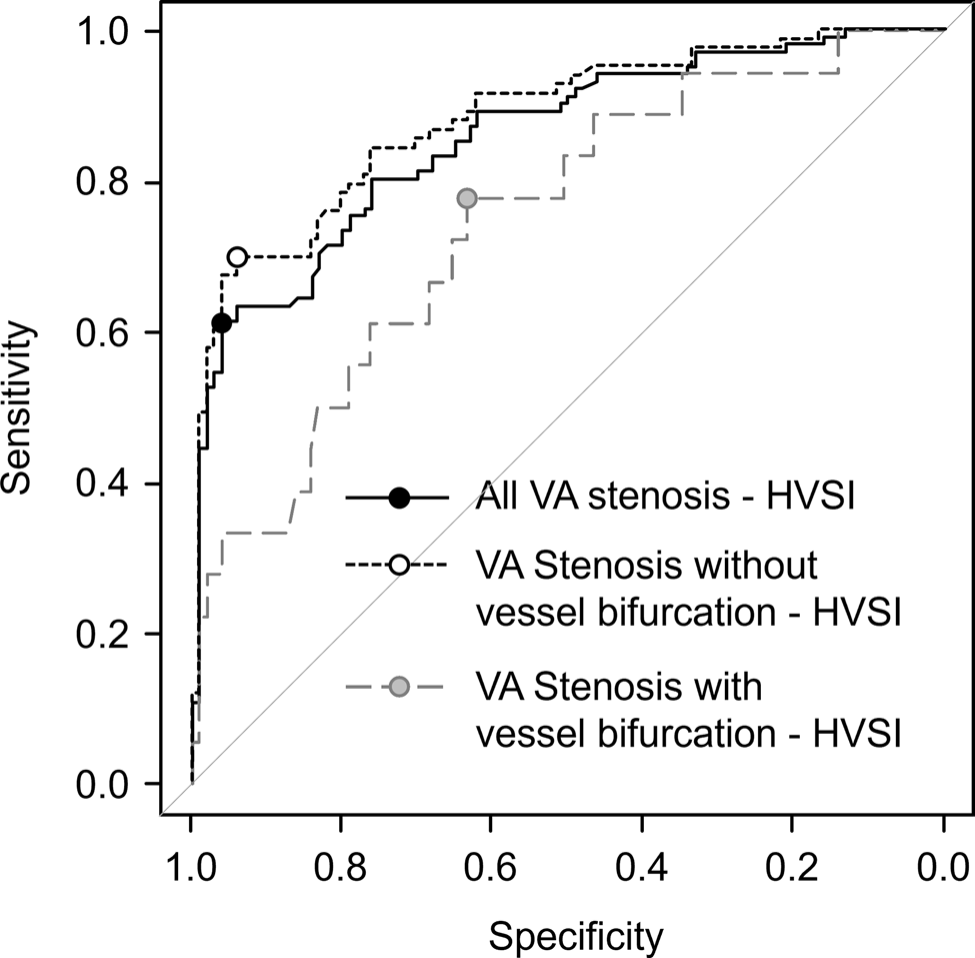
ROC curves for HVSI indicating vascular access stenosis requiring VAIVT, stratified by presence or absence of vessel bifurcation

ROC curves for the HVSI at the anastomosis indicating the need for VAIVT are shown in Fig. 5. When all VA stenoses were considered positive, the HVSI cutoff was 104.5, with a sensitivity of 61.4%, specificity of 96.0%, and AUC of 0.86. When stenosis cases without bifurcation were considered positive, the HVSI cutoff was 119.5, with a sensitivity of 69.9%, specificity of 94.1%, and AUC of 0.88. When stenosis cases with bifurcation were considered positive, the HVSI cutoff was 262.0 with a sensitivity of 77.8%, specificity of 63.4%, and AUC of 0.75. Diagnostic performance tended to decrease in the presence of bifurcation.

In multivariable logistic regression analyses adjusting for clinically relevant covariates including AVF location/type and other baseline differences, higher HVSI values were associated with lower odds of low FV and stenosis/VAIVT indication. This directionality is clinically consistent with the HVSI concept. Higher HVSI reflects higher access blood flow and lower downstream resistance, whereas lower HVSI reflects reduced flow and higher resistance.

After multivariable adjustment for relevant clinical covariates including AVF type/location, HVSI remained independently associated with (i) VA stenosis/VAIVT indication (Table 3) and (ii) low brachial artery flow volume (Table 4), with higher HVSI values consistently indicating a lower likelihood of stenosis and low flow.

Verification results for reproducibility of the HVSI cutoffs in the additional cohort are shown in Table 2. In the verification cohort, diagnostic performance for detecting FV < 400 mL/min using the predefined cutoff was: sensitivity 66.7%, specificity 94.1%, positive predictive value (PPV) 66.7%, and negative predictive value (NPV) 94.1%. For detecting stenosis/VAIVT indication, sensitivity and specificity were both 100.0%, with PPV 100.0% and NPV 100.0%. Concordance between the HVSI-based judgment and the ultrasound-based judgment was 18/20 (90.0%) for FV < 400 mL/min and 19/20 (95.0%) for VAIVT indication. Because this verification cohort was small, these findings should be interpreted as exploratory and hypothesis-generating.

**Table 2:** Verifying the reproducibility of HVSI cutoff values

Number	Age [year]	Gender	Anastomosis type	Vessel bifurcation	Flow volume [mL/min]	Resistance index	Flagged by HVSI cutoff	HVSI	Matching (FV-HVSI)	Matching (VAIVT-HVSI)
1	73	Female	AVF	No	668	0.48	Yes	71	No	Yes
2	73	Male	AVF	Yes	207	0.73	Yes	61	Yes	Yes
3	81	Male	AVF	No	896	0.57	No	845	Yes	Yes
4	82	Female	AVF	No	866	0.41	No	675	Yes	Yes
5	57	Female	AVF	No	926	0.62	No	217	Yes	Yes
6	58	Male	AVF	No	715	0.59	No	486	Yes	Yes
7	79	Female	AVF	No	609	0.62	No	200	Yes	Yes
8	60	Female	AVF	No	1032	0.44	No	686	Yes	Yes
9	70	Male	AVF	No	1200	0.53	No	600	Yes	Yes
10	81	Female	AVF	No	469	0.49	No	298	Yes	Yes
11	58	Male	AVF	No	755	0.53	No	881	Yes	Yes
12	46	Male	AVF	Yes	1341	0.41	No	698	Yes	Yes
13	66	Female	AVF	No	924	0.47	No	498	Yes	Yes
14	67	Male	AVF	No	781	0.51	No	920	Yes	Yes
15	75	Male	AVF	No	983	0.54	No	575	Yes	Yes
16	69	Male	AVF	Yes	160	0.75	Yes	41	Yes	Yes
17	54	Male	AVF	No	1274	0.34	No	839	Yes	Yes
18	73	Male	AVF	No	405	0.59	No	299	Yes	Yes
19	81	Female	AVF	No	261	0.69	Yes	122	No	No
20	62	Male	AVF	No	3025	0.39	No	999	Yes	Yes

Verification cohort consisted of ultrasound-confirmed normal VA cases; ‘Yes’ indicates that the HVSI value fell below the predefined cutoff, not that VAIVT was required

## Discussion

This study demonstrated that the Hemodialysis Vascular Sound Index (HVSI), measured at a single standardized location (the anastomosis), is a practical, non-invasive indicator associated with ultrasound-derived parameters and capable of identifying patients likely to have reduced access flow or clinically significant stenosis requiring intervention.

Importantly, the association between HVSI and both VA stenosis/VAIVT indication and low FV remained significant after multivariable adjustment (Tables 3 and 4) for potential confounders, suggesting that HVSI provides information beyond baseline clinical characteristics and AVF type (Tables 3 and 4). Because HVSI is scaled such that higher values correspond to higher access flow, the inverse association observed in the logistic models is clinically expected.

**Table 3:** Multivariable logistic regression for VA stenosis (study group vs. control)

Variable	Adjusted OR (95% CI)	p value
**HVSI (per 100 units)**	**0.44 (0.33–0.57)**	<0.001
Age (years)	0.98 (0.95–1.02)	0.316
Male	0.89 (0.40–1.95)	0.764
Dialysis duration (years)	0.99 (0.94–1.04)	0.662
Diabetes mellitus	1.15 (0.54–2.45)	0.717
CRP (mg/dL)	0.92 (0.75–1.13)	0.445
Past VAIVT (yes)	2.87 (1.18–6.97)	0.020
AVF type: wrist (vs. brachial)	0.29 (0.08–0.96)	0.043
AVF type: anatomical snuffbox (vs. brachial)	1.30 (0.32–5.28)	0.718

Outcome: VA stenosis (1 = stenosis/study group), Predictor of interest: HVSI (per 100-unit increase), Adjusted for: age, sex, dialysis duration, diabetes, CRP, past VAIVT, AVF type

**Table 4:** Multivariable logistic regression for low flow volume (FV < 400 mL/min)

Variable	Adjusted OR (95% CI)	p value
**HVSI (per 100 units)**	**0.07 (0.03–0.17)**	<0.001
Age (years)	1.01 (0.97–1.05)	0.681
Male	0.31 (0.10–0.96)	0.043
Dialysis duration (years)	1.00 (0.93–1.06)	0.892
Diabetes mellitus	1.03 (0.37–2.91)	0.954
CRP (mg/dL)	0.95 (0.72–1.25)	0.715
Past VAIVT (yes)	7.16 (1.70–30.19)	0.007
AVF type: wrist (vs. brachial)	0.78 (0.17–3.66)	0.750
AVF type: anatomical snuffbox (vs. brachial)	4.81 (0.67–34.72)	0.120

Outcome: FV < 400 mL/min (1 = yes), Predictor of interest: HVSI (per 100-unit increase), Adjusted for: same covariates as above

A significant positive correlation was observed between HVSI and brachial artery flow volume (R^2^ = 0.58, *p* < 0.001), supporting the concept that HVSI reflects access blood flow. HVSI quantifies vascular sounds using frequency-domain analysis designed to capture flow-related acoustic energy. Turbulence at the anastomosis generates vascular murmurs, and changes in flow velocity and vessel wall properties influence the spectral components of the recorded signal. Previous work has suggested that filtering higher-frequency components may improve correlation with flow volume, and the present study supports the clinical utility of this approach.

In ROC analyses, HVSI showed strong performance for detecting reduced FV, with AUCs of 0.90–0.94 across FV thresholds of 500, 400, and 350 mL/min. These results suggest that HVSI may serve as a convenient screening metric to identify patients who should undergo confirmatory ultrasound evaluation. In addition, the HVSI threshold for overall VA stenosis/VAIVT indication (cutoff 104.5) achieved high specificity (96.0%), which may be useful for triaging. Importantly, diagnostic performance differed according to the presence of vessel bifurcation between the anastomosis and stenosis. Bifurcation likely provides collateral flow that partially compensates for stenosis, reducing the functional impact on flow volume and thereby diminishing diagnostic accuracy based solely on sound-derived indices. Therefore, while HVSI appears most reliable in non-bifurcated segments, values in the intermediate range may warrant careful interpretation and ultrasound confirmation.

Regarding reproducibility, the additional verification cohort supported the feasibility of applying predefined HVSI cutoffs to a separate sample. However, because the cohort size was limited (*n* = 20), these data should be interpreted as exploratory. Larger studies assessing inter- and intra-operator variability under controlled and real-world conditions are needed. In this study, HVSI measurements were performed by trained clinical engineers, but the measurement itself is simple: if the diaphragm is placed over the anastomosis with appropriate contact, recordings can be obtained within seconds. Therefore, the procedure may be feasible for routine use in clinical settings after standardized training and protocol optimization, including control of background noise and consistent contact pressure.

The present design enrolled clinically significant stenosis cases requiring VAIVT and compared them with ultrasound-confirmed stable controls. This approach resembles a case-control diagnostic accuracy framework and may overestimate correlations and AUC values relative to an unselected, consecutively enrolled real-world population. Accordingly, the results should be interpreted as reflecting performance under controlled conditions in clearly defined groups. Future multicenter studies using consecutively enrolled cohorts are necessary to confirm generalizability and determine how HVSI performs as a screening adjunct across a broader disease spectrum.

This work also highlights the importance of considering differences in baseline characteristics, such as AVF location and prior VAIVT history. Although matching was used, residual imbalance remained, and multivariable models were used to mitigate confounding. Because HVSI is a functional index reflecting flow, it may retain practical value across AVF locations; however, additional analyses by access site and anatomical subtype may further refine cutoffs or improve interpretability.

## Conclusion

Thus, HVSI should be interpreted not as a replacement for vascular ultrasound, but as a simple screening adjunct to triage patients who require confirmatory ultrasound and/or VAIVT. In this prospective matched observational study, HVSI showed high diagnostic accuracy for reduced FV and clinically significant VA stenosis, supporting its potential role in routine VA surveillance. Because the study design and selected comparison groups may overestimate diagnostic performance compared with consecutively enrolled real-world cohorts, further validation in diverse populations—particularly in consecutively enrolled multicenter cohorts—is warranted.

## Electronic supplementary material

Below is the link to the electronic supplementary material.

Supplementary Material 1

Supplementary Material 2

Supplementary Material 3

Supplementary Material 4

Supplementary Material 5

Supplementary Material 6

Supplementary Material 7

## Data Availability

All minimal data, the reproducibility dataset, the data dictionary, and the changelog are publicly available on Figshare (DOI: 10.6084/m9.figshare.29999032).

## References

[1] Campos RP, Chula DC, Perretto S, et al. Accuracy of physical examination and intra-access pressure in the detection of stenosis in hemodialysis arteriovenous fistula. Semin Dial. 2008;21:269–73. 10.1111/j.1525-139X.2007.00419.x. 18248519 10.1111/j.1525-139X.2007.00419.x

[2] Maldonado-Cárceles AB, García-Medina J, Torres-Cantero AM. Performance of physical examination versus ultrasonography to detect stenosis in hemodialysis arteriovenous fistula. J Vasc Access. 2017;18:30–34. 10.5301/jva.5000616. 27834455 10.5301/jva.5000616

[3] Roca-Tey R, Samon R, Ibrik O, Roda E, González-Oliva JC, Martínez-Cercós R, et al. Five years of vascular access stenosis surveillance by blood flow rate measurements during hemodialysis using the Delta-H method. J Vasc Access. 2012;13(3):321–28. 10.5301/jva.5000053. 22287222 10.5301/jva.5000053

[4] Roca-Tey R, Ibeas J, Moreno T, Gruss E, Merino JL, Vallespín J, et al. Spanish multidisciplinary vascular access group (GEMAV). Dialysis arteriovenous access monitoring and surveillance according to the 2017 Spanish guidelines. J Vasc Access. 2018;19(5):422–29. 10.1177/1129729818761307. 29544403 10.1177/1129729818761307

[5] Wang H-Y, Wu C-H, Chen C-Y, Lin B-S. Novel noninvasive approach for detecting arteriovenous fistula stenosis. IEEE Trans Biomed Eng. 2014;61:1851–57. 10.1109/TBME.2014.2308906. 24845295 10.1109/TBME.2014.2308906

[6] Malindretos P, Liaskos C, Bamidis P, Chryssogonidis I, Lasaridis A, Nikolaidis P. Computer-assisted sound analysis of arteriovenous fistula in hemodialysis patients. Int J Artif Organs. 2014;37:173–76. 10.5301/ijao.5000262. 24619890 10.5301/ijao.5000262

[7] Mansy HA, Hoxie SJ, Patel NH, Sandler RH. Computerised analysis of auscultatory sounds associated with vascular patency of haemodialysis access. Med Biol Eng Comput. 2005;43:56–62. 10.1007/BF02345123. 15742720 10.1007/BF02345123

[8] Kawai M, Suzuki A. Analysis and evaluation of vascular sounds in hemodialysis. Proceedings of the 35th IEEE Engineering in Medicine and Biology Society (EMBC). Osaka, Japan: 2013 July 3–7: 1017–23. doi: 10.1109/EMBC.2013.6609676. 10.1109/EMBC.2013.6609676 24109863

[9] Presta P, Carullo N, Armeni A, et al. Evaluation of arteriovenous fistula for hemodialysis with a new generation digital stethoscope: a pilot study. Int Urol Nephrol. 2024;56:1763–71. 10.1007/s11255-023-03895-5. 38093038 10.1007/s11255-023-03895-5

[10] Zhou G, Chen Y, Chien C, et al. Deep learning analysis of blood flow sounds to detect arteriovenous fistula stenosis. NPJ Digit Med. 2023;6:163. 10.1038/s41746-023-00894-9. 37658233 10.1038/s41746-023-00894-9 PMC10474109

[11] Tsuboi M, Suzuki H, Kawai H, Ejima T, Mitsuishi F. Vascular sound visualization system is useful for monitoring and surveillance of vascular access. J Vasc Access. 2022;23:390–97. 10.1177/1129729821993984. 33586508 10.1177/1129729821993984

[12] Hitomi Y. Verification of whether the “electronic auscultation HVSI value (hemodialysis vascular sound index)” using shunt sound algorithm analysis can be used as a treatment intervention standard, such as the flow volume (FV) measured by an echo device. Jpn J Dial Ther. 2024;57(5):227–28. (In Japanese). doi: 10.4009/jsdt.57.227.

[13] Kukita K, Ohira S, Amano I, et al. 2011 update Japanese Society for dialysis therapy guidelines of vascular access construction and repair for chronic hemodialysis. Ther Apher Dial. 2015;19(Suppl 1):1–39. 10.1111/1744-9987.12296. 25817931 10.1111/1744-9987.12296

[14] Shinzato T, Nakai S, Fujita Y, et al. Determination of Kt/V and protein catabolic rate using pre- and postdialysis blood urea nitrogen concentrations. Nephron. 1994;67:280–90. 10.1159/000187980. 7936017 10.1159/000187980

[15] Kanda Y. Investigation of the freely available easy-to-use software ‘EZR’ for medical statistics. Bone Marrow Transpl. 2013;48:452–58. 10.1038/bmt.2012.244. 10.1038/bmt.2012.244 PMC3590441 23208313

